# Anti-Inflammatory Effect of *Cinnamomum japonicum* Siebold’s Leaf through the Inhibition of p38/JNK/AP-1 Signaling

**DOI:** 10.3390/ph16101402

**Published:** 2023-10-03

**Authors:** Ji Min Kim, In A Jung, Jae Min Kim, Moon-Hee Choi, Ji Hye Yang

**Affiliations:** 1 College of Korean Medicine, Dongshin University, Naju 58245, Republic of Korea; jeemin3004@naver.com (J.M.K.); ina6014@naver.com (I.A.J.); kjm4994@naver.com (J.M.K.); 2 Department of Biochemical Engineering, College of Engineering, Chosun University, Gwangju 61452, Republic of Korea; 3 Sumsumbio Co., Ltd., Jangseong-gun 57248, Republic of Korea

**Keywords:** *Cinnamomum japonicum* Siebold’s leaf, radical scavenging, anti-inflammatory, p38/JNK/AP-1 signaling

## Abstract

*Cinnamomum japonicum* Siebold (CJ) branch bark, commonly known as Japanese cinnamon, has been used for various culinary and medicinal applications for many centuries. Although the efficacy of CJ branch bark’s anti-inflammatory and antioxidant activity for the treatment of various diseases has been confirmed, the efficacy of CJ leaves (CJLs) has not been examined. We therefore investigated whether CJL3, an ethyl acetate extract of a 70% ethanol CJL extract, exerts anti-inflammatory effects on lipopolysaccharide (LPS)-activated Kupffer cells, specialized macrophages found in the liver. Liver inflammation can activate Kupffer cells, inducing the release of pro-inflammatory molecules that contribute to tissue damage. We found that CJL3 has high 2,2-diphenyl-1-picrylhydrazyl and 2,2-azino-bis (3-ethylbenzthiazoline-6-sulfonic acid) radical-scavenging activity. Among the CJL extracts, CJL3 exhibited the greatest polyphenol content, with protocatechuic acid and 4-hydroxybenzoic acid being the most abundant. In addition, we verified that CJL3, which has strong antioxidant properties, ameliorates LPS-induced pro-inflammatory responses by inhibiting p38/JNK/AP-1 signaling. CJL3 therefore has potential for treating liver disease, including hepatitis.

## 1. Introduction

Kupffer cells, specialized macrophages found in the liver, play a crucial role in maintaining liver health and function and are involved in both protective and inflammatory responses within the liver [1]. In healthy individuals, Kupffer cells help clear pathogens, toxins, and debris from the bloodstream [2] and contribute to the immune response by recognizing and engulfing foreign particles and microorganisms [3]. In addition, Kupffer cells produce various cytokines, chemokines, and inflammatory mediators that regulate immune responses and coordinate liver responses to injury and infection. However, in liver diseases, such as hepatitis, alcoholic liver disease, non-alcoholic fatty liver disease, and liver fibrosis, Kupffer cells may exhibit imbalanced activity [4]. Liver inflammation can activate Kupffer cells, inducing the release of pro-inflammatory molecules that contribute to tissue damage. Chronic inflammation and Kupffer cell activation therefore promote the progression of liver disease [5]. Regulating Kupffer cells in liver disease may therefore facilitate the development of targeted therapies. 

*Cinnamomum japonicum* Siebold (CJ) is an evergreen tree belonging to the *Lauraceae* family [6]. CJ is well adapted to temperate climates and can be found growing in the wild in various countries, including Korea, China, and Japan. It has leathery, oblong, dark-green leaves and smooth, grayish-brown bark. The branch bark, commonly known as Japanese cinnamon, has an aromatic flavor and has been used for various culinary and medicinal applications for many centuries [7]. Modern pharmacological studies have demonstrated that cinnamon exhibits beneficial properties, including antioxidant, anti-inflammatory, and antimicrobial activity, blood sugar regulation, potential cardiovascular benefits, and cognitive function and neuroprotective effects [8,9,10,11]. In a previous study, we found that CJ bark exhibits greater antioxidant activity than the leaves, and CJ bark have good anti-inflammatory effect [12]. However, the leaves had a greater extract yield than the bark, which is a great advantage in developing natural therapeutics. We therefore investigated the potential of *C. japonicum* Siebold’s leaves for development as drug material for liver diseases such as hepatitis. We previously obtained various solvent extracts of CJ leaves (CJLs), including hot water, 70% ethanol (EtOH), and 100% EtOH extracts, as well as extracts obtained via ultrasonication, confirmed the antioxidant activities, and determined the total polyphenol and flavonoid content [13]. The 70% EtOH extract exhibited the greatest antioxidant activity, as well as the greatest total phenolic and flavonoid content. In this study, we found that among the CJL extracts, CJL3 (an ethyl acetate extract of the 70% EtOH CJL extract) had the greatest radical-scavenging activity, as well as the highest polyphenol content. Furthermore, CJL3 exerted anti-inflammatory effects on lipopolysaccharide (LPS)-activated Kupffer cells by inhibiting the p38/JNK/AP-1 signaling pathway, suggesting that it may be a potential candidate for the treatment of inflammatory liver disease.

## 2. Results

### 2.1. Antioxidant Activity and Phenolic Content of CJL Extracts

**Figure 1:**
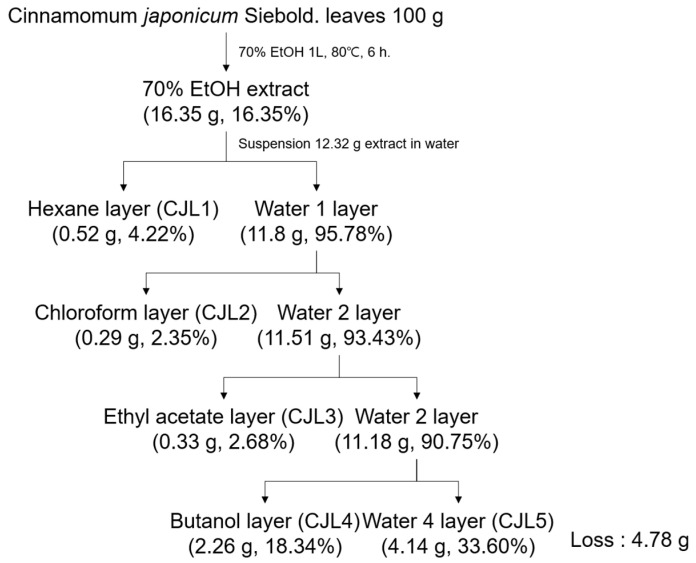
Extraction and fractionation of *Cinnamomum japonicum* Sieb. leaves.

**Figure 2:**
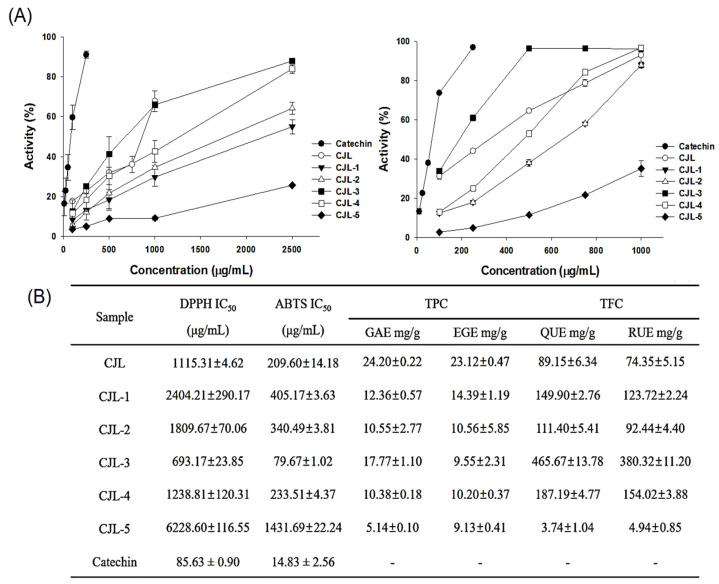
(**A**) Antioxidant activity of the 70% ethanol extract (CJL) and fraction samples (CJL1–5) toward 2,2-diphenyl-1-picrylhydrazyl (DPPH) and 2,2-azino-bis (3-ethylbenzthiazoline-6-sulfonic acid) (ABTS). (**B**) Total polyphenol content (TPC) and total flavonoid content (TFC) of *Cinnamomum japonicum* Sieb. leaves (CJLs) (GAE: Gallic acid equivalent, EGE: Ethyl gallate equivalent, QUE: Quercetin equivalent, RUE: Rutin equivalent).

We collected fractionated samples (CJLs and CJL1–5) using sequential extraction and separation (Figure 1) and determined their 2,2-diphenyl-1-picrylhydrazyl (DPPH) and 2,2-azino-bis (3-ethylbenzthiazoline-6-sulfonic acid) (ABTS) radical-scavenging activities. CJL3 exhibited IC_50_ values of 693.17 μg/mL for DPPH and 79.67 μg/mL for ABTS, indicating high activity. We also determined the polyphenol and flavonoid content of the 70% ethanol extract (CJLs) and fractionated samples (CJL1–5) (Figure 2B). Gallic acid, ethyl gallate, quercetin, and rutin were used as standards. As a result, we found that the total phenolic content expressed as ethyl gallate equivalent (17.77 mg/g), and the total flavonoid content expressed as quercetin equivalent (465.67 mg/g) and rutin equivalent (380.32 mg/g) in the ethyl acetate layer (CJL3), were higher than in the other layers (Figure 2).

### 2.2. Identification of CJL3 Components

**Figure 3:**
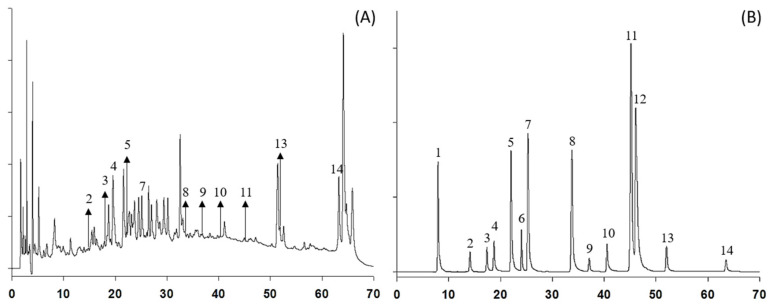
High-performance liquid chromatography (HPLC) profile of (**A**) CJL3 and (**B**) standard mixture using diode array detection at 280 nm. (1) Gallic acid; (2) Catechin; (3) Epigallocatechin gallate; (4) (-)-Epicatechin; (5) Ethyl gallate; (6) Rutin; (7) *p*-Coumaric acid; (8) Coumarin; (9) Cinnamyl alcohol; (10) Quercetin; (11) Cinnamyl aldehyde; (12) *trans*-Cinnamic acid; (13) Eugenol; (14) Cinnamyl acetate.

To identify the polyphenols in CJL3, high-performance liquid chromatography (HPLC) was performed with gallic acid, catechin, epigallocatechin gallate, (-)-epicatechin, ethyl gallate, rutin, p-coumaric acid, coumarin, cinnamyl alcohol, quercetin, cinnamyl aldehyde, trans-cinnamic acid, eugenol, and cinnamyl acetate as standards (Supplementary Data). Cinnamyl acetate, epicatechin, catechin, and p-coumaric acid were the most abundant polyphenols (Figure 3 and Table 1). Previous studies reported coumarin, cinnamic acid, and cinnamyl alcohol in 70% ethanol extracts, as well as cinnamic acid and cinnamaldehyde in 100% ethanol extracts, of plants of the same species as cinnamon [14,15]. Our results are comparable to those of previous studies; however, we identified additional flavan-3-ol compounds, including catechin and quercetin.

**Figure 4:**
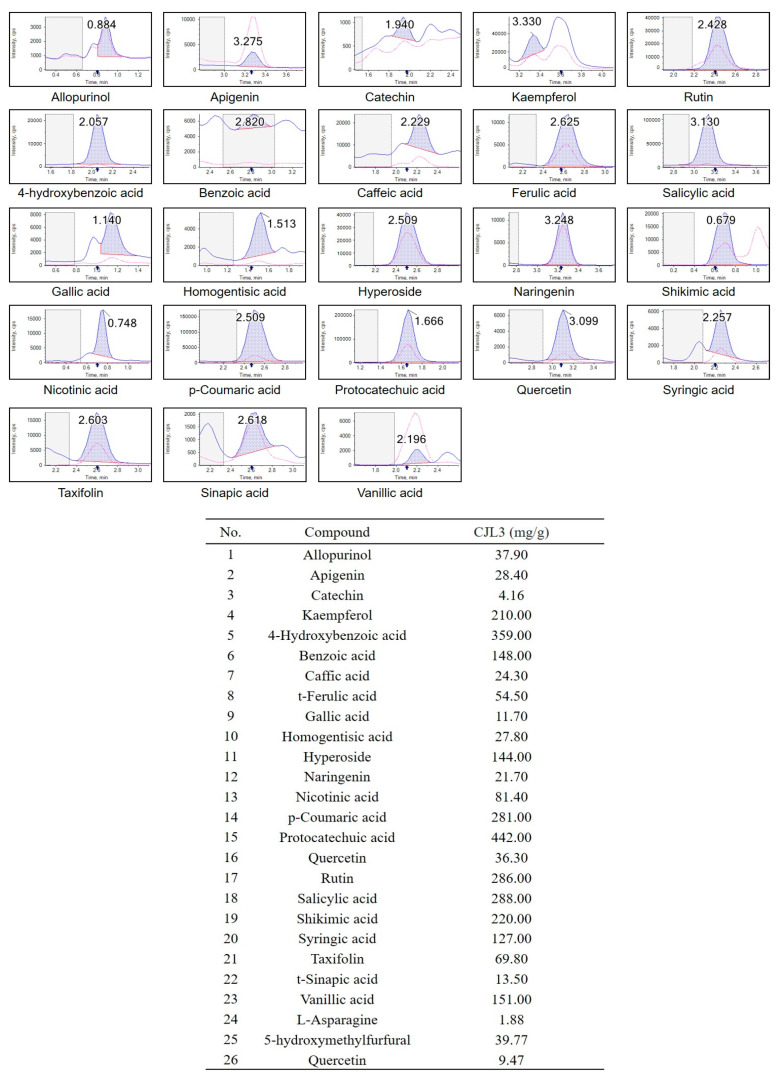
Polyphenols identified in CJL3 and quantified using LC-MS/MS.

LC-MS/MS was used to quantitatively analyze the polyphenols in CJL3. Among the 26 analyzed polyphenols (Supplementary Data), protocatechuic acid (PCA), 4-hydroxybenzoic acid, salicylic acid, rutin, and p-coumaric acid were the most abundant (Figure 4). PCA was the most abundant (442.00 µg/g). Our LC-MS/MS results differed from the HPLC results.

### 2.3. Suppression of Nitric Oxide (NO) and Pro-Inflammatory Cytokine Production by CJL3 

**Figure 5:**
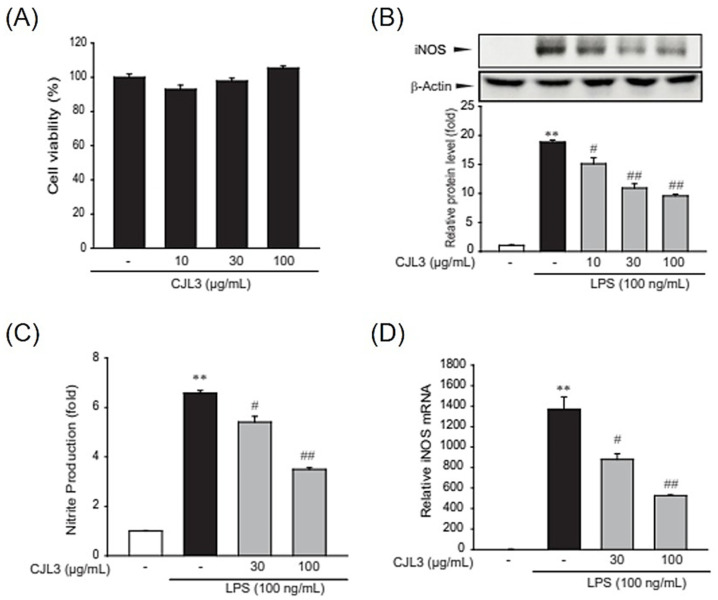
(**A**) The cytotoxicity of CJL3 in immortalized mouse Kupffer cells (ImKCs). Cells were treated with various concentrations of CJL3 (10–100 μg/mL) for 24 h, and cell viability was determined using MTT assays. (**B**–**D**) Inhibitory effect of CJL3 on LPS-induced nitric oxide (NO) production and nitric oxide synthase (iNOS) expression in ImKCs. (**B**) iNOS expression in LPS-activated ImKCs. Cells were pre-treated with CJL3 (10–100 μg/mL) for 1 h and then incubated with 100 ng/mL LPS for 12 h. iNOS protein levels in the cell lysates were determined using western blot (**C**) NO production in LPS-activated ImKCs. Cells were treated with CJL3 (30–100 μg/mL) and/or LPS for 15 h, and NO production was measured using the Griess reagent. (**D**) iNOS mRNA levels were determined using real-time polymerase chain reaction (RT-PCR). Cells were pre-treated with CJL3 (30–100 μg/mL) for 1 h and then incubated with 100 ng/mL LPS for 6 h. Data are presented as the mean ± standard error of the mean of three replicates; ** *p* < 0.01, significant versus vehicle-treated control; ^##^ *p* < 0.01, ^#^ *p* < 0.05, significant versus LPS alone.

**Figure 6:**
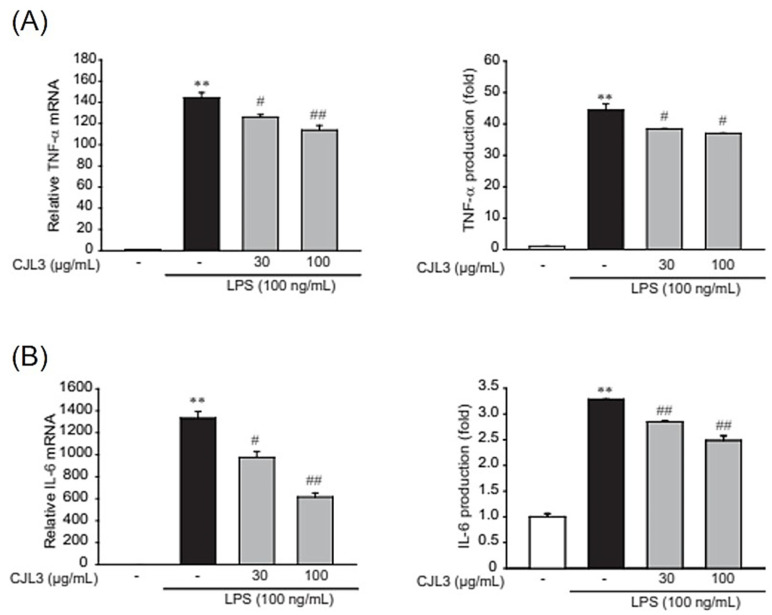
Inhibitory effect of CJL3 on LPS-induced pro-inflammatory cytokine expression in immortalized mouse Kupffer cells. (**A**) Tumor necrosis factor (TNF)-α and (**B**) interleukin (IL)-6 levels. Cells were treated with 30 μg/mL or 100 μg/mL CJL3 for 1 h and subsequently incubated with LPS for 6 h. TNF-α and IL-6 mRNA levels were determined using real-time polymerase chain reaction (RT-PCR) assays (**left**) and enzyme-linked immunosorbent assays (**right**). Data are presented as the mean ± standard error of the mean of three replicates; ** *p* < 0.01, significant versus vehicle-treated control; ^##^ *p* < 0.01, ^#^ *p* < 0.05, significant versus LPS alone.

To evaluate the cytotoxicity of CJL3 in an immortalized mouse Kupffer cell line (ImKC), we assessed cell viability at various CJL3 concentrations (10, 30, and 100 μg/mL). There was no significant difference in cell viability between the vehicle-treated group and treatment with up to 100 μg/mL CJL3 (Figure 5A). Next, we investigated the suppression of nitric oxide (NO) production by CJL3. NO, which is produced by inducible nitric oxide synthase (iNOS), has been implicated as an inflammatory mediator. Under normal physiological conditions, NO exerts anti-inflammatory effects, whereas overproduction under abnormal conditions induces inflammation. We found that CJL3 inhibits iNOS expression (Figure 5B) and LPS-induced NO production (Figure 5C). Furthermore, we investigated whether CJL3 suppresses iNOS transcription and confirmed that CJL3 reduces LPS-induced iNOS mRNA levels in ImKCs (Figure 5D). Next, we investigated the inhibitory effect of CJL3 on pro-inflammatory cytokine production. Pro-inflammatory cytokines, such as tumor necrosis factor (TNF)-α and interleukin (IL)-6, play essential roles in the development of various inflammatory liver diseases that result in liver cirrhosis [16]. We found that pretreatment with CJL3 significantly reduces TNF-α and IL-6 mRNA levels and that CJL3 inhibits LPS-induced cytokine (TNF-α and IL-6) release (Figure 6). These results indicate that CJL3 ameliorates LPS-induced production of NO and pro-inflammatory cytokines. 

### 2.4. Inhibition of the LPS-Induced p38/Jun N-Terminal Kinase (JNK)/AP-1 Signaling Pathway by CJL3

**Figure 7:**
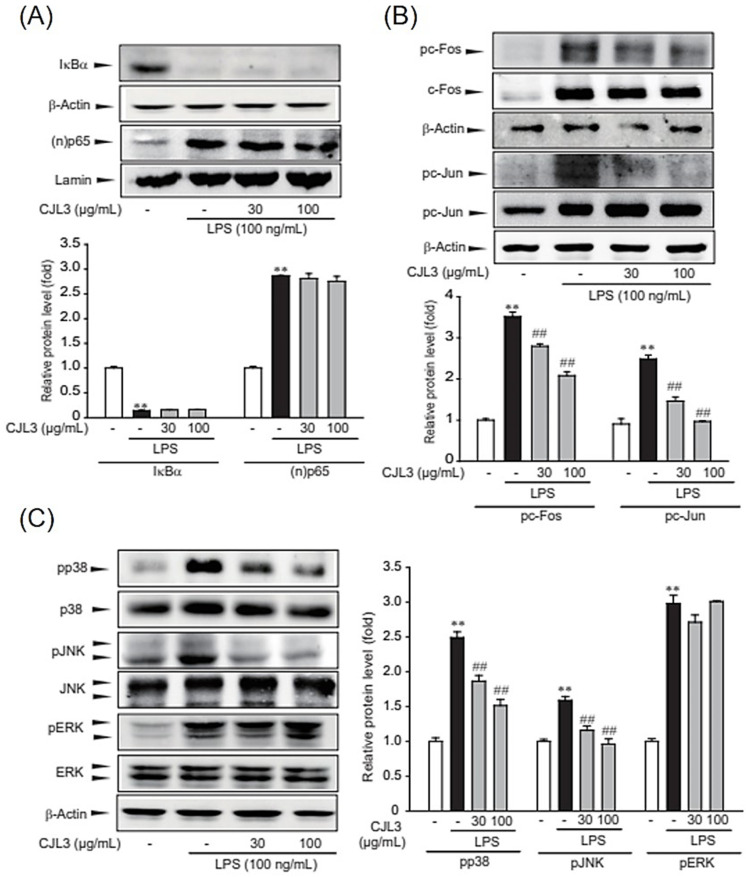
CJL3-induced inhibition of AP-1 in LPS-activated ImKCs. (**A**) Immunoblotting for IκBα and (n)p65. ImKCs were pre-treated with CJL3 (30–100 μg/mL) for 1 h and then stimulated with 100 ng/mL LPS for 15 min (IκBα) or 3 h ((n)p65). (**B**) Immunoblotting for c-Jun and c-Fos phosphorylation. Cells were pre-treated with CJL3 (30–100 μg/mL) for 1 h prior to LPS stimulation for 2 h. (**C**) Immunoblotting for mitogen-activated protein kinase (MAPK) phosphorylation. Cells were pre-treated with CJL3 (30–100 μg/mL) for 1 h prior to LPS stimulation for 30 min. Data are represented as the mean ± standard error of the mean of three replicates; ** *p* < 0.01, significant versus vehicle-treated control; ^##^ *p* < 0.01, significant versus LPS alone.

To determine whether the p38/JNK /AP-1 signaling pathway is suppressed by CJL3, we investigated whether CJL3 blocks LPS-induced degradation of IκBα, nuclear translocation of p65, and/or phosphorylation of c-Fos/c-Jun in ImKCs. IκBα inhibits the NF-κB complex by binding to and sequestering it in the cytoplasm; p65 is the major transcriptional activation domain within the NF-κB complex; and c-Fos/c-Jun proteins are the most widely studied AP-1 components. NF-κB and AP-1 are transcription factors that play crucial roles in inflammation, immunity, cell proliferation, and apoptosis [17,18]. We found that LPS increases IκBα degradation, p65 nuclear translocation, and c-Fos /c-Jun phosphorylation and that CJL3 pretreatment inhibits c-Fos/c-Jun phosphorylation (Figure 7A,B). 

We investigated whether mitogen-activated protein kinase (MAPK) phosphorylation is involved in CJL3-mediated inhibition of AP-1 signaling (c-Fos/c-Jun phosphorylation). We found significantly increased ERK1/2, p38, and c-JNK phosphorylation following LPS treatment of naïve control cells, whereas p38 and JNK phosphorylation was suppressed in CJL3-treated cells (Figure 7C). These data suggest that CJL3 ameliorated LPS-induced pro-inflammatory responses by inhibiting p38/JNK/AP-1 signaling. 

## 3. Discussion

Inflammation is a normal response of the immune system to injury or infection and is a key component of the defense mechanism against various liver diseases. However, a prolonged or excessive inflammatory response can contribute to liver damage and disease [19]. During liver injury, Kupffer cells release inflammatory substances, such as cytokines and reactive oxygen species (ROS), which can further damage liver cells and contribute to the development of liver disease [20]. We previously compared *C. japonicum* Siebold’s bark and leaves and found that the bark exhibits greater antioxidant activity than the leaves. However, the extract yield of the leaves is greater than that of the bark, which is a great advantage for developing natural therapeutics [12]. We therefore investigated the potential of CJL3 as a drug for suppressing inflammation during liver damage using ImKCs.

In this study, we found that the DPPH and ABTS radical-scavenging activities of CJL3 are higher than those of the other fraction samples (CJL1–5). Previous studies have reported that the 70% ethanol extract of *C. cassia* Blume, which belongs to the same genus as Japanese cinnamon, has an IC_50_ value of 0.89 ± 0.22 mg/mL in the DPPH assay. Considering the unit conversion of approximately 890 g/mL, this confirmed the superior antioxidant activity of *C. cassia* Blume branches to that of the 70% ethanol extract of *C. cassia* Blume branches and leaves [21]. In another study, a 50% ethanol extract of *C. cassia* Blume exhibited an antioxidant activity of approximately 90% at 100 μg/mL in the DPPH assay [22]. In this study, we found that the scavenging activity of the bark extract was approximately 82% and that of the leaf extract was approximately 35% (Figure 2). 

We analyzed the polyphenols in CJL3 using HPLC for screening and LC-MS/MS for quantitative analysis and obtained contradictory results (Figure 4). This may be attributed to the HPLC analyses comparing retention time with a standard, and peaks eluting at the same time may contain multiple substances. In contrast, LC-MS/MS analysis first separates the substances using HPLC, and each component is subsequently qualitatively and quantitatively analyzed using mass spectrometry. The HPLC/MS analysis was calculated from the similarity index data between the standard and sample UV spectra. As it is not a simple separation analysis, after HPLC analysis, LC-MS/MS analysis is performed for accurate analysis. 

LC-MS/MS analysis of 26 polyphenols confirmed that PCA (442.00 µg/g) and 4-hydroxybenzoic acid (359.00 µg/g) are the major components in CJL3. Both PCA and 4-hydroxybenzoic acid are anthocyanin metabolites. A recent study comparing the anti-inflammatory efficacy of PCA and 4-hydroxybenzoic acid reported that although both 4-hydroxybenzoic acid and PCA effectively target ROS-induced toxicity, only PCA targets inflammation [23]. PCA has also shown promising anti-inflammatory activity in different rat models (carrageenan-induced paw edema, cotton pellet-induced granuloma, and Freund’s adjuvant arthritis) of inflammation [24]. PCA is widely distributed in nature and is structurally similar to the antioxidant compounds gallic, caffeic, vanillic, and syringic acids [25]. There are no previous reports of the anti-inflammatory activity of 4-hydroxybenzoic acid, a single component derived from *C. japonicum* Siebold’s leaf; therefore, the researchers’ findings are significant [7]. Our results therefore suggest that the antioxidant and anti-inflammatory effects of CJL3 may be caused by various polyphenol components in CJL3, including PCA and 4-hydroxybenzoic acid.

Next, we investigated whether CJL3 inhibits the inflammatory response in ImKCs. ROS serve as signaling molecules and regulate immune cell activation, migration, and function during inflammation [26]. ROS contribute to the elimination of pathogens by damaging their genetic material and cellular components [27]. Although ROS play a crucial role in the immune response, excessive or dysregulated ROS production can lead to oxidative stress, and prolonged or excessive oxidative stress can cause cellular lipid peroxidation, protein oxidation, and DNA damage [26], which contribute to inflammation and are associated with various inflammatory diseases. Moreover, ROS can activate redox-sensitive signaling pathways, such as MAPKs, NF-κB, and AP-1, leading to the production of pro-inflammatory cytokines, chemokines, and other inflammatory mediators [28]. NF-κB is one of the most important transcription factors that activates the inflammatory response. In the inactive state, NF-κB is sequestered in the cytoplasm by IκBs. It is activated in response to inflammation or infection and controls the migration of inflammatory cells and the production of inflammatory molecules and increases blood vessel permeability [17,29]. AP-1 is a transcription factor involved in cell proliferation and differentiation. It is activated by inflammation or infection and enhances the development and proliferation of inflammatory cells. The c-Jun/c-Fos proteins form an AP-1 complex [30], are activated by inflammation or infection, and promote the growth and proliferation of inflammatory cells. MAPKs, which include extracellular signal-regulated kinases (ERKs), p38, and c-Jun N-terminal kinase [31], are cell signaling pathways that play an important role in cell growth, death, and division [32]. Numerous studies have shown that extracts exert anti-inflammatory activity by inhibiting NF-κB/AP-1/MAPK signals [33,34]. Our results indicated that CJL3 specifically blocks LPS-induced AP-1/p38/JNK signaling. 

## 4. Materials and Methods

### 4.1. Chemical Extracts of Cinnamomum japonicum Sieb. Leaf

In April 2021, *Cinnamomum japonicum* Sieb. leaves were collected from Wando Arboretum (Wando, Korea) in Jeollanam-do, washed, dried with hot air (40 °C), stored, and then pulverized for use. *C. japonicum* Sieb. leaves were collected from the arboretum located in Wando of Jeollanam-do Forest Resources Research institute (126° 9885.42″ E longitude and 34° 3154.30″ N latitude) according to the Wild Plant Protection Act of Korea. The morphological identification of the specimen was performed at National Institute of Biological Resources Information System (Specimen number: NIBRVP00000017321). The leaves were washed, dried with hot air (40 °C), stored, and then pulverized for use. Approximately 100 g dried *C. japonicum* Sieb. leaves were added to 1 L 70% ethanol and extracted at 80 °C for 6 h [35]. The extract was filtered, concentrated using a reduced-pressure concentrator, and freeze-dried. The concentrated 70% ethanol extract (CJLs) was dissolved in 50% methanol and placed in a fractional funnel. Hexane was added and shaken vigorously, and the supernatant was collected after the layers separated. The supernatant was returned to the funnel, shaken with hexane, and the supernatant collected. This process was repeated 2–3 times to obtain a concentrated supernatant (CJL1). The concentrated supernatant was dissolved in distilled water and placed in a separatory funnel, and chloroform was added for further separation (CJL2). After chloroform-layer separation, ethyl acetate (EtOAc) was added to the supernatant and the layers separated (CJL3). Butanol was added to the EtOAc supernatant for further separation (CJL4 and CJL5). The separated extracts were filtered, concentrated using a reduced-pressure concentrator, and freeze-dried (Figure 1).

### 4.2. Antioxidative Activities and Phenolic Content

#### 4.2.1. DPPH Radical-Scavenging Activity

DPPH radical-scavenging activity was assessed as previously described [36], with modifications. Briefly, *C. japonicum* Sieb. leaf extracts were prepared at concentrations of 0–2500 μg/mL, and 800 μL 0.25 mM DPPH reagent was mixed with 200 μL of each concentration of CJLs. The reaction was carried out in the dark for 30 min, and the absorbance measured at 517 nm using a Biotek Synergy HT multidetection microplate reader. Catechin was used as the positive control.

#### 4.2.2. ABTS Radical-Scavenging Activity

The ABTS radical-scavenging assay was performed as previously described [37], with some modifications. Briefly, 7 mM ABTS reagent and 2.45 mM potassium persulfate were mixed in a 1:1 ratio and allowed to react in the dark for 12 h to generate ABTS radicals. Approximately 1 mL ABTS radical reagent was added to 200 μL of each concentration of *C. japonicum* Sieb. leaf extract. The reaction was carried out in the dark for 30 min, and the absorbance measured at 730 nm using a Biotek Synergy HT multidetection microplate reader. Catechin was used as the positive control.

#### 4.2.3. TPC and TFC

TPC was determined using a modified Folin–Ciocalteu method [38]. *C. japonicum* Sieb. leaf extract was mixed with 500 μL 0.2 M Folin–Ciocalteu phenol reagent (Sigma Aldrich, St. Louis, MO, USA) and 500 μL 2% sodium carbonate aqueous solution (*w*/*v*), allowed to react in the dark for 30 min, and the absorbance measured at 750 nm using a BioTek Synergy HT multidetection microplate reader. The final concentration of the extract was 500 μg/mL. The total phenolic content was expressed as gallic acid (GAE) mg/g equivalent and ethyl gallate (EGE) mg/g equivalent based on the black curve.

TFC was determined using the method described by Eom et al. [39]. Approximately 1.5 mL methanol was added to 500 μL *C. japonicum* Sieb. leaf extract, followed by 100 μL 10% aluminum chloride, 100 μL 1 M potassium acetate, and 2.8 mL distilled water. The reaction was carried out at room temperature for 40 min, and absorbance measured at 415 nm using a BioTek Synergy HT multidetection microplate reader. The final concentration of the extract was 500 μg/mL. The TFC was expressed as quercetin (QUE) mg/g equivalent and rutin (RUE) mg/g equivalent based on calibration curves.

### 4.3. High-Performance Liquid Chromatography with Diode Array Detection (HPLC–DAD) Analysis

For the quantitative analysis of polyphenols in the EtOAc fraction (CJL3), HPLC (SPD-20A; SHIMADZU Co., Japan) was used to identify the active ingredients. A C18 column (Shim-pak GIS-ODS, 5 μm, 250 × 4.6 mm) and a C18 Guard column (XTerra™ RP18 3.5 μm) were used. Acetic acid (0.02%) in water (A) and acetic acid (0.02%) in acetonitrile (B) were used as the mobile phases. The gradient conditions of the mobile phase were 0 min, 10% (B); 0–60 min, 50% (B); 60–65 min, 10% (B); 65–70 min, 10% (B). Analyses were performed at room temperature with a 20 μL sample injection volume and monitored at 280 nm. The following 14 polyphenols were used as standards: gallic acid, catechin, epigallocatechin gallate, (-)-epicatechin, ethyl gallate, rutin, p-coumaric acid, coumarin, cinnamylalcohol, quercetin, cinnamylaldehyde, trans-cinnamic acid, eugenol, and cinnamyl acetate. All chemicals and reagents were purchased from Sigma Aldrich (St. Louis, MO, USA) and were HPLC grade.

### 4.4. Quantitative Analysis of Polyphenols Using HPLC MS/MS

The LC-MS/MS analyses were performed using an AB SCIEX 4000 Q Trap LC/MS/MS System (Shimadzu LC 20A System, Kyoto, Japan). Formic acid (0.1%) in water (A) and formic acid (0.1%) in acetonitrile (B) were used as the mobile phases. The gradient conditions of the mobile phase were 0–0.1 min, 5% (B); 0.1–2 min, 40% (B); 2–3 min, 80% (B); 3–5 min, 80% (B); 5–5.1 min, 5% (B); 8 min stop. Using Turbo Ion Spray, MS/MS analyses were performed in both negative and positive modes. 

### 4.5. Cell Culture

ImKCs were obtained from Sigma-Aldrich (St. Louis, MO, USA) and grown in Dulbecco’s modified Eagle’s medium (high glucose) supplemented with 50 U/mL penicillin/streptomycin and 10% fetal bovine serum at 37 °C in a humidified 5% CO_2_ atmosphere. Cells were passaged after reaching 90% confluence, detached with a cell scraper, and subcultured in a 1:5 ratio in 100 mm dishes. Cells were cultured continuously from the third to fifteenth passage. Cells were frozen every fifth passage starting from the third passage. Cells (2 × 10^5^ cells/mL) were then seeded on plates and incubated for 24 h. Thereafter, it was exchanged with medium containing different concentrations of CJL3 (10, 30, and 100 μg/mL) or LPS (100 ng/mL) as a positive control.

### 4.6. MTT Assays

Cells were plated in 48-well plates and treated with chemicals for 24 h. Cell viability was determined by MTT staining (0.2 mg/mL, 4 h), as previously described [40]. The media in the wells were removed and the produced formazan crystals were dissolved by adding 200 μL dimethyl. Absorbance was measured at 540 nm using a microplate reader (Spectramax, Molecular Devices, Sunnyvale, CA, USA). Cell viability was defined relative to the untreated control [viability (% control) = 100 × (absorbance of treated sample)/(absorbance of control]). 

### 4.7. Analysis of Nitric Oxide Production

Cells were plated in 12-well plates and treated with CJL3 and/or LPS for 15 h. A standard curve was constructed using sodium nitrite (Sigma, St. Louis, MO, USA), and the nitrite content of the culture medium was determined by mixing the samples with Griess reagent (Sigma, St. Louis, MO, USA), as previously described [40]. After incubation for 30 min, absorbance was measured at 548 nm using an ELISA microplate reader (Spectramax, Molecular Devices).

### 4.8. Immunoblot Analysis

Protein extraction, subcellular fractionation, sodium dodecyl sulfate-polyacrylamide gel electrophoresis, and immunoblot analyses were performed according to previously published procedures [41]. Briefly, samples were separated using 7.5% gel electrophoresis and transferred to nitrocellulose membranes. The nitrocellulose membranes were then incubated with the indicated primary antibody, followed by incubation with horseradish peroxidase-conjugated secondary antibody. Immunoreactive proteins were visualized using ECL chemiluminescence detection (Amersham Biosciences, Buckinghamshire, UK). Equal protein loading was monitored, and the integrity of subcellular fractionation was verified using β-actin immunoblotting. Antibodies against iNOS and IκBα were provided by Santa Cruz Biotechnology (Dallas, TX, USA). Phospho-ERK1/2, ERK1/2, phospho-p38, p38, phospho-JNK1/2, JNK1/2, phospho-c-Jun, c-Jun, phospho-c-Fos, c-Fos, lamin, and phospho-IκBα were obtained from Cell Signaling (Danvers, MA, USA). Horseradish peroxidase-conjugated goat anti-rabbit, anti-mouse, and anti-goat antibodies were purchased from Invitrogen (Carlsbad, CA, USA). Actin antibody was purchased from Sigma-Aldrich (St. Louis, MO, USA).

### 4.9. Enzyme-Linked Immunosorbent Assay (ELISA)

Cytokine (TNF-α and IL-6) levels in the culture medium were measured using ELISA kits. TNF-α and IL-6 levels were measured using an Invitrogen analyzer (Carlsbad, CA, USA).

### 4.10. RT-PCR Analysis

Total RNA was extracted using TRIzol reagent (Invitrogen, Carlsbad, CA, USA) according to the manufacturer’s instructions. To obtain cDNA, total RNA (2 µg) was reverse-transcribed using an oligo(dT)_16_ primer. The cDNA was amplified using a high-capacity cDNA synthesis kit (Bioneer, Daejeon, Korea) and a thermal cycler (Bio-Rad, Hercules, CA, USA). Real-time PCR (RT-PCR) was performed with STEP ONE (Applied Biosystems, Foster City, CA, USA) using SYBR Green premix according to the manufacturer’s instructions (Applied Biosystems). Primers were synthesized by Bioneer (Table 2).

### 4.11. Statistical Analysis

One-way analysis of variance was used to assess the statistical significance of differences among treatment groups. For statistically significant differences, the Newman–Keuls test was used for comparisons between multiple groups. Data were expressed as the mean ± standard error of the mean.

## 5. Conclusions

In conclusion, we found that CJL3, an ethyl acetate extract of 70% EtOH CJLs, exhibits greater antioxidant activity than the other CJL extracts. Furthermore, we identified PCA as the most abundant polyphenol in CJL3. We found that CJL3 ameliorates LPS-induced production of NO and pro-inflammatory cytokines by inhibiting p38/JNK/AP-1 signaling, thus attenuating LPS-induced pro-inflammatory responses. We therefore propose that CJL3 may be a promising candidate for treating liver disease, including hepatitis. Based on these results, future studies to investigate the effects of CJL3 in animal models of liver disease, including hepatitis, fibrosis, and cirrhosis, are warranted.

## Figures and Tables

**Table 1:** Polyphenols identified in CJL3 and quantified using high-performance liquid chromatography.

No.	Compound	Retention Time	CJL3 (Unit: μ/g)
1	Gallic acid	-	-
2	Catechin	14.102	237.19 ± 32.57
3	Epigallocatechin gallate	17.522	191.84 ± 30.77
4	(-)-Epicatechin	18.631	1238.46 ± 275.57
5	Ethyl gallate	22.051	145.78 ± 6.17
6	Rutin	-	
7	*p*-Coumaric acid	24.981	210.94 ± 99.94
8	Coumarin	34.907	3.96 ± 4.93
9	Cinnamylalcohol	36.909	191.53 ± 7.23
10	Quercetin	40.617	88.69 ± 39.45
11	Cinnamylaldehyde	45.263	11.14 ± 4.68
12	*trans*-Cinnamic acid	-	-
13	Eugenol	51.993	196.07 ± 39.43
14	Cinnamyl acetate	63.396	5261.47 ± 247.13

**Table 2:** Sequence for primers used in real-time quantitative PCR assays.

Gene		Sequence (5′-3′)
mouse iNOS	sense	5′-CCTCCTCCACCCTACCAAGT-3′
	antisense	5′-CACCCAAAGTGCTTCAGTCA-3′
mouse TNF-α	sense	5′-AAGCCTGTAGCCCACGTCGTA-3′
	antisense	5′-AGGTACAACCCATCGGCTGG-3′
mouse IL-6	sense	5′-TCCATCCAGTTGCCTTCTTG-3′
	antisense	5′-TTCCACGATTTCCCAGAGAAC-3′
mouse GAPDH	sense	5′-TGCCCCCATGTTTGTGATG-3′
	antisense	5′-TGTGGTCATGAGCCCTTCC-3′

## Data Availability

All data are presented in the article.

## Supplementary Materials

The following supporting information can be downloaded at: https://www.mdpi.com/article/10.3390/ph16101402/s1, supplementary data: The compound structures confirmed by HPLC and LC-MS/MS data.
