# Supplementary material for: Anti-Inflammatory Effect of Cinnamomum japonicum Siebold’s Leaf through the Inhibition of p38/JNK/AP-1 Signaling
Source: Pharmaceuticals (Basel). 2023 Oct 3;16(10):1402. doi: 10.3390/ph16101402 (PMC10610235; doi:10.3390/ph16101402)

Supplementary Data. The compound structures confirmed by HPLC and LC-MS/MS data

1. HPLC data compound structures.

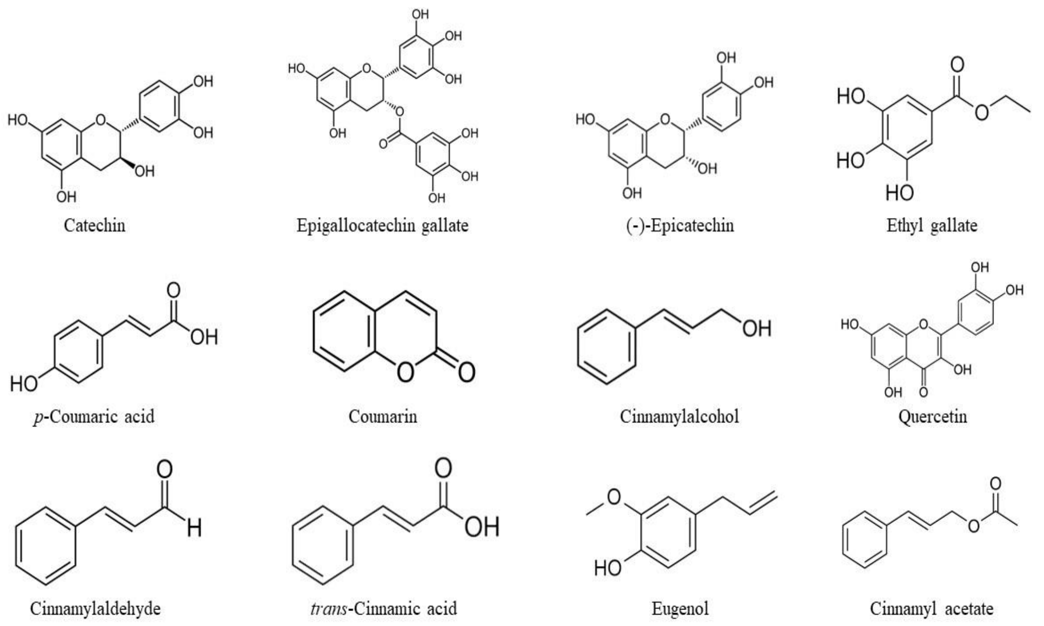

2. LC-MS/MS data compound structures

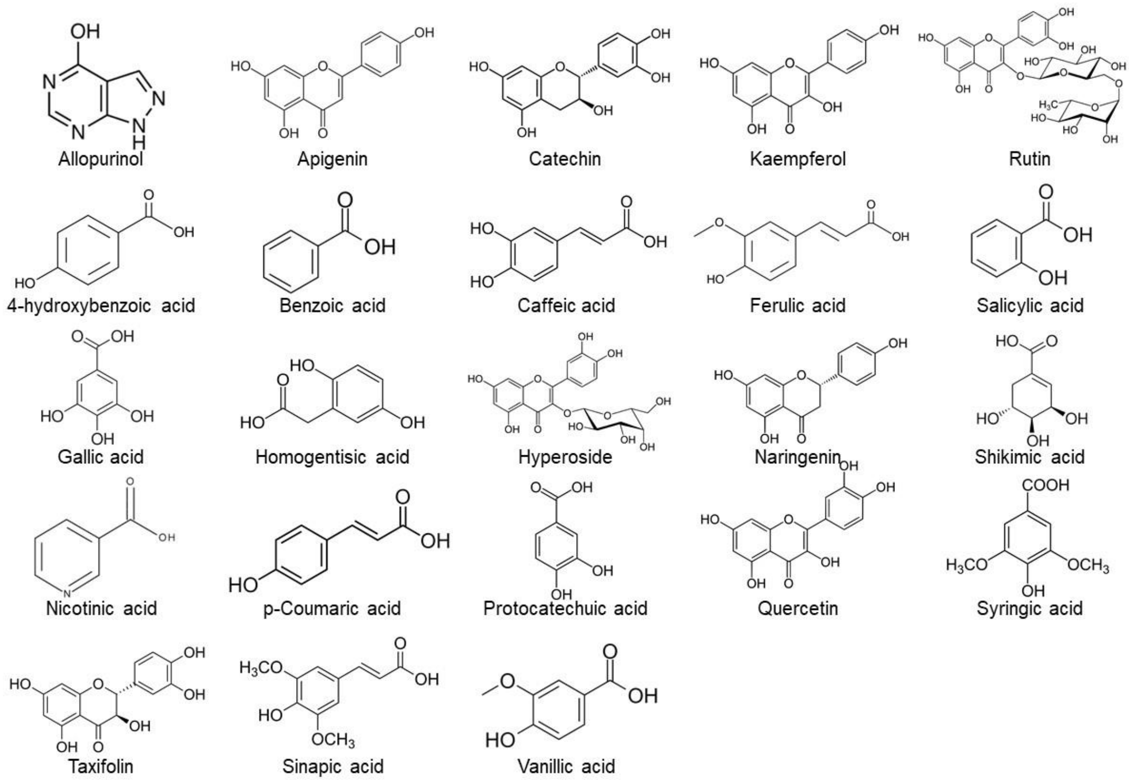

Supplement: Supplementary file 1 [file pharmaceuticals-16-01402-s001.zip › pharmaceuticals-2584477-supplementary.pdf]
